# Switching Hypnotic Drugs to Remimazolam and Antagonizing With Flumazenil: A Rapid Method for Ending General Anesthesia

**DOI:** 10.7759/cureus.78108

**Published:** 2025-01-27

**Authors:** Rebecca Koch, Hielke Markerink, Richard Witkam, Jörgen Bruhn, Lucas Van Eijk

**Affiliations:** 1 Anesthesiology, Pain and Palliative Medicine, Radboud University Medical Center, Nijmegen, NLD

**Keywords:** emergence from anesthesia, flumazenil, remimazolam, switch and antagonize, triple anesthesia

## Abstract

The emergence from general anesthesia is currently difficult to predict and may be accompanied by respiratory complications. Switching all hypnotic drugs to remimazolam at the end of the operation and antagonizing it with flumazenil might lead to a rapid, smooth, and safe patient awakening, potentially increasing operating theater efficiency and reducing costs. In this case series of five patients, anesthesia with a combination of remimazolam, propofol, and sevoflurane was transitioned to remimazolam at 0.9-1.0 mg/kg/h as the sole hypnotic agent near the end of the operation. Subsequently, remimazolam was antagonized with 0.5 mg flumazenil. This approach resulted in a rapid, predictable, and smooth emergence and recovery, free from excitation or hemodynamic and respiratory disturbances. Additionally, postoperative opioid requirements were minimal, and no anti-emetic medication was necessary. The authors conclude that the "switch and antagonize" concept is feasible and promising, warranting further evaluation and refinement in the near future.

## Introduction

Ending anesthesia after an operation is typically achieved by discontinuing all anesthetic agents and allowing them to wear off until the patient is deemed sufficiently awake and/or spontaneously breathing for extubation. Predicting the time required for anesthetic agents to wear off adequately can be challenging, potentially leading to prolonged emergence from anesthesia and delayed extubation, thereby occupying the costly resources of an operating theater and staff. Furthermore, during the transitional phase between full general anesthesia and full wakefulness, the patient is at risk of respiratory complications such as laryngospasm, bronchospasm, and respiratory depression, which may result in serious injury to the patient [1].

In contrast to the significant emphasis placed on preventing complications during intubation, the potential risks associated with emergence and post-extubation are often underestimated [2]. Moreover, waiting for all anesthetic agents to wear off completely can prolong the patient's stay in the recovery room and is associated with more post-anesthetic recovery issues, including hypotension, respiratory depression, postoperative nausea and vomiting (PONV), and patient discomfort, all of which are caused by the residual effects of the anesthetic agents used.

With the introduction of the short-acting benzodiazepine remimazolam for general anesthesia, it has become possible to antagonize its effects with flumazenil immediately after the operation is completed. Using remimazolam as the sole hypnotic for maintaining general anesthesia may offer advantages, such as reduced intraoperative hypotension [3], but it also has disadvantages, including less suppression of intraoperative movements compared to other hypnotics [4,5], the risk of residual sedation in some patients [6], a higher likelihood of postoperative nausea [7], and a poorer overall quality of recovery when used as a single sedative [6].

Therefore, the use of remimazolam for anesthesia maintenance may require further optimization before being incorporated into standard anesthesia regimens. This could involve combining it with the favorable properties of propofol (anti-emetic effects) and volatile anesthetics (suppression of spinal reflexes). Employing a standard maintenance strategy that combines propofol, a volatile anesthetic, and remimazolam also has the benefit of a rapid emergence time, as each hypnotic can be administered at a low dose and cleared through different mechanisms. Another potential approach is to switch all hypnotic drugs to remimazolam toward the end of the operation, offering the advantage of its antagonism with flumazenil.

Recently, a case report has described the use of remimazolam after general anesthesia with sevoflurane, a volatile anesthetic, followed by flumazenil administration [8]. In this report, the patient was extubated under deep anesthesia, and flumazenil was administered after extubation. Additionally, Jeon et al. [9] reported the transition from propofol to remimazolam one hour before the end of surgery, followed by flumazenil reversal in 54 adults with mental disabilities undergoing dental treatment under general anesthesia.

A systematic review and meta-analysis has demonstrated that the combination of remimazolam and flumazenil accelerates recovery from general anesthesia and reduces the risk of respiratory depression compared to the commonly used hypnotic drug propofol [10]. Switching all hypnotic drugs to remimazolam at the end of surgery, followed by antagonization with flumazenil, may facilitate a fast and smooth awakening of the patient. Furthermore, this anesthetic regimen could enhance patient and physician satisfaction, improve patient safety, and increase operating theatre efficiency, thereby reducing costs. This technique is anticipated to be increasingly adopted in daily clinical practice. Here, we report our experience transitioning to remimazolam followed by flumazenil in five orthopedic patients undergoing total hip arthroplasty.

## Case presentation

The Medical Research Ethics Committee of East Netherlands approved this retrospective case series, which used only data already stored in the patients' electronic files without further interventions or measurements (approval number: 2024-17800). Only data from patients who explicitly consented to the anonymous use of all their clinical data for retrospective research were included, as documented in their electronic patient records. The Committee waived the requirement for additional patient consent.

Patient information

We retrospectively evaluated adult patients aged <70 years, with an American Society of Anesthesiologists (ASA) classification <3 and body mass index (BMI) <30, who were scheduled for total hip prosthesis surgery under general anesthesia with remimazolam and received its antagonist, flumazenil, at the end of the surgery. Using these criteria, a search of the electronic patient record system identified five patients. Demographic characteristics are presented in Table 1.

**Table 1:** Demographic characteristics M: male, F: female, BMI: body mass index, ASA: American Society of Anesthesiologists

	Patient 1	Patient 2	Patient 3	Patient 4	Patient 5	Mean	SD
Sex (M/F)	F	M	M	F	F	-	-
Age	69	55	83	42	41	58	14.4
Length (cm)	161	180	180	165	167	170.6	7.52
Weight (kg)	70	76	78	70	70	72.8	3.36
BMI	27	23.6	24	25.6	25.1	25.06	1.008
ASA class	2	2	3	2	2	2.2	0.32

Five adult patients (two males and three females) with a mean age of 58 ± 14.4 years were included. Four patients were classified as ASA physical status class II and one as ASA class III. All patients had normal BMI (25 ± 1.0).

Anesthetic regime and switching and antagonizing

Next to the standard non-invasive monitoring, including electrocardiography, oxygen saturation, and non-invasive blood pressure, a frontal electroencephalogram (EEG) was attached to the patient’s forehead to measure the bispectral index (BIS). A neuromuscular monitor was also attached to the patient’s wrist to assess the degree of neuromuscular block.

During induction, infusion pumps delivering propofol (approximately 2 mg/kg/h) and remimazolam (approximately 0.3 mg/kg/h) were initiated and administered through the same intravenous line. Subsequently, a bolus of 15-20 µg sufentanil and remimazolam (0.20-0.21 mg/kg) were administered. All doses were calculated based on actual body weight. The intraoperative medications administered, including time frames for switching and antagonizing, are summarized in Table 2.

**Table 2:** Perioperative medication administered and time frames of switch and antagonize MAC: minimum alveolar concentration

	Patient 1	Patient 2	Patient 3	Patient 4	Patient 5
For induction					
Sufentanil (µg)	15	20	15	15	20
Remimazolam (mg/kg)	0.2	0.2	0.21	0.2	0.21
After induction					
Piritramide (mg)	10	20	10	10	10
For maintenance					
Propofol (mg/kg/h)	2	2	2	2	2
Remimazolam (mg/kg/h)	0.3	0.3	0.29	0.3	0.3
Sevoflurane (MAC)	0.4	0.4	0.4	0.4	0.4
After switching: remimazolam (mg/kg/h)	0.9	0.9	1.0	1.0	1.0
Time (min) from increasing remimazolam (switch) to stop remimazolam	24	20	22	43	22
Time (min) from flumazenil administration to extubation	2	2	1	1	2
Postoperative analgesia					
Recovery: piritramide (mg)	5	0	5	5	0
Ward: oxycodone (mg) in the first 24 hours		0	5	0	0
Ward: buprenorphine sublingual (mg) (allergic to oxycodone)	0.2				

When the patient was asleep, a single dose of 35-40 mg of rocuronium was administered to induce muscle relaxation for the placement of the endotracheal tube. Sevoflurane was added at approximately end-tidal 0.4 MAC, age-corrected. This MAC value was displayed directly on the monitor of the GE Datex Ohmeda Advance anesthesia machine. The age-corrected MAC for sevoflurane was internally calculated using the following formula:

\[ \text{MAC}_{\text{age}} = 2.05 \cdot 1.32 \cdot 10^{-0.00303 \cdot \text{age (years)}} \]

This combination of remimazolam, propofol, and sevoflurane has recently been reported as triple anesthesia [11] and is commonly used in our department. After induction, 10 mg of piritramide was added in four patients, and 20 mg of piritramide was added in one patient as a long-acting opioid. No other opioids were administered after the induction of anesthesia.

As is standard in our department for orthopedic surgery, patients received premedication with 1,000 mg of paracetamol and 150 mg of pregabalin. Intraoperatively, a multimodal analgesic concept was employed, consisting of 40 mg of parecoxib, 8 mg of dexamethasone, a 5-10 mg bolus of esketamine followed by a continuous infusion at 5-10 mg/h, and a 40 mg/kg bolus of magnesium chloride followed by a continuous infusion at 500 mg/h. Approximately 45 minutes before the end of surgery, esketamine and magnesium chloride were discontinued. When the end of surgery was anticipated within 20-25 minutes, propofol and sevoflurane were stopped, and remimazolam was increased to 0.9-1.0 mg/kg/h (Table 2). After confirming the absence of residual neuromuscular blockade, spontaneous breathing was encouraged. After the operation, the infusion of remimazolam was stopped, and 0.5 mg of flumazenil was administered 1-2 minutes later. Once the patients opened their eyes, the endotracheal tube was removed.

Clinical parameters and time frames after antagonizing

**Figure 1:**
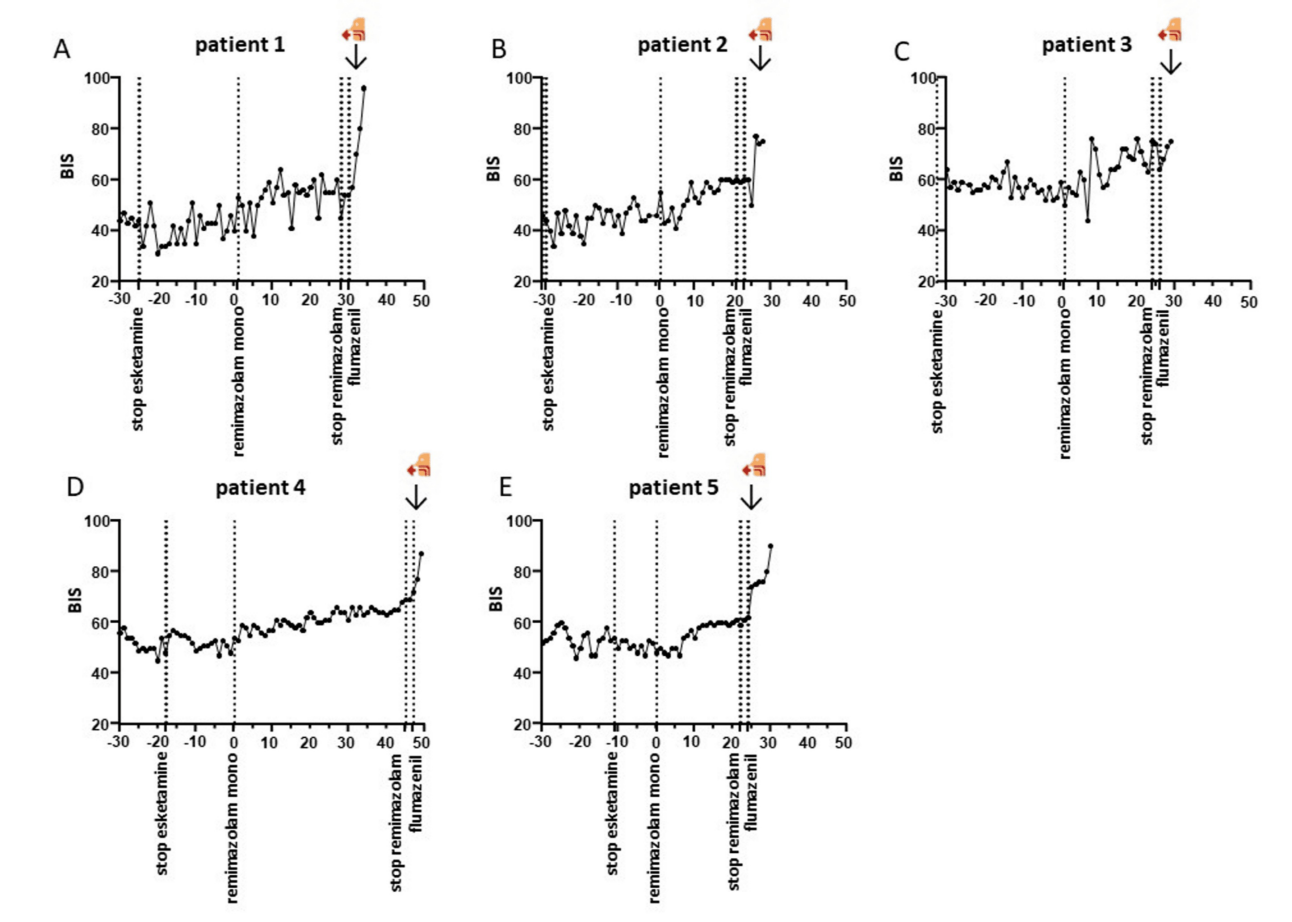
BIS values in switching and antagonizing The X-axis displays time in minutes, with the moment of switching to remimazolam as a mono-anesthetic set at T=0. BIS values during the switching and antagonizing phases are shown. Panels A–E represent patients 1–5. The moment of extubation is represented by the logo of a face with (or without) a tube BIS: bispectral index

All patients remained hemodynamically stable throughout the procedure. No significant alterations in blood pressure were observed after switching to remimazolam as a mono-anesthetic. The time from flumazenil administration to extubation was two minutes in three patients and one minute in two. Additionally, no desaturation occurred in any of the patients. BIS values increased from 67.2 ± 9 to 83.3 ± 6.8 following the administration of flumazenil (Figure 1).

No signs of excitation were observed in any of the patients, and no sudden changes in vital parameters occurred after antagonization. No patient movements or coughing were observed during switching or antagonization. Patients emerged calmly from general anesthesia and were quickly transferred to the recovery room. Overall, no adverse events occurred.

Clinical course in the recovery room and normal ward

The administration of opioids in the recovery room and normal ward was minimal, as presented in Table 2. In the recovery room, three patients received a dose of 5 mg piritramide, while no piritramide was administered to two patients. On the ward, one patient with an allergy to oxycodone received a single sublingual dose of 0.2 mg buprenorphine. Among the other four patients, one received a single 5 mg dose of oxycodone during the first 24 hours postoperatively, while the remaining three patients did not receive any additional opioids on the ward. No anti-emetic drugs were required during the entire 24-hour postoperative period.

## Discussion

In this case series, a concept of "switch and antagonize" was used, which involved switching from a combination of remimazolam, propofol, and sevoflurane as hypnotic maintenance of anesthesia to remimazolam as the sole hypnotic agent near the end of the operation. Combined with flumazenil, this approach resulted in a rapid, predictable, and smooth recovery free from excitation or hemodynamic and respiratory disturbances. It demonstrates the feasibility of achieving controlled emergence from general anesthesia in humans through specific antidotes.

In 2023, Araki and Inoue described switching to remimazolam after anesthesia with sevoflurane, followed by flumazenil for the deep extubation of a patient with bronchial asthma [8]. While they administered flumazenil after deep extubation, Jeon et al. [9] reported an approach more similar to ours, involving the administration of flumazenil prior to extubation. They described converting from propofol to remimazolam with flumazenil reversal in adults with mental disabilities undergoing dental treatment one hour before the end of surgery [10]. To our knowledge, there are no other reports on "switch and antagonize."

Several interesting questions remain. When should the switch occur? The timing of the switch should be long enough to sufficiently decrease the residual amounts of sevoflurane and/or propofol. However, a switch as close as possible to the end of surgery would be advantageous from a practical standpoint. Our data suggest that the one hour prior to the end of surgery, as used in the study by Jeon et al., may not be necessary. Even in the presence of long-acting opioids, a time point 20-30 minutes before the end of surgery might be sufficient. Nevertheless, this should be further investigated with larger sample sizes. It is also unclear whether an even shorter time interval could be feasible.

Certain operations, such as hip arthroplasty, allow for a better estimation of the remaining procedure time compared to others, such as intraoral procedures (Jeon et al. reported a relatively high standard deviation for the duration of remimazolam infusion at 64.7 ± 25 minutes) or laparoscopic surgery. This variability should be considered when planning the timing of the switch for specific procedures.

Another anesthetic regimen that could be interesting to investigate in larger randomized trials is propofol, remimazolam, and sevoflurane. At the end of the surgery, hypercapnic hyperventilation could be employed to rapidly eliminate sevoflurane and propofol, along with flumazenil, for the antagonism of remimazolam. This strategy has shown promise in several small studies [12-14].

How to switch? Araki and Inoue repeated the induction dose of remimazolam while switching. However, we do not believe this is necessary. Even after stopping propofol and/or sevoflurane, the plasma levels require some time to decrease, which might correspond to the time needed for the plasma levels of remimazolam to increase during initiation or escalation of remimazolam infusion. This aligns with the study by Jeon et al., who also did not administer a bolus of remimazolam during switching.

Araki and Inoue used a remimazolam dose of 0.8 mg/kg/h from the completion of the laparoscopic procedure until skin closure. This is comparable to our dosing regimen of 0.9 mg/kg/h after switching. Jeon et al. reported a remimazolam dose of 1-2 mg/kg/h, adjusted based on a processed EEG value after switching.

How much flumazenil is needed to antagonize remimazolam? Many studies on remimazolam and flumazenil, such as Toyota et al. [15], used a standard dose of 0.5 mg of flumazenil. Jeon et al. and our study also used the same dose of 0.5 mg of flumazenil. In contrast, Araki and Inoue used a dose of 0.9 mg of flumazenil. A titration approach, such as administering 0.2 mg of flumazenil followed by repeated doses of 0.1 mg every minute up to 1 mg until arousal [6], is also an option. However, as fast extubation without a prolonged "twilight zone" between full general anesthesia and complete wakefulness is preferred, starting with a higher standard dose might be more advantageous. Greater pharmacodynamic-pharmacokinetic insight into the relationship between remimazolam plasma levels and the required flumazenil dose could provide clinically important information in the future.

Some potentially negative clinical aspects of the remimazolam-flumazenil setting should be considered, such as extubation recall, PONV, and postoperative pain. Sato et al. concluded in a study of 163 patients that the incidence of extubation recall after remimazolam anesthesia with flumazenil antagonism during emergence did not significantly differ from that after propofol anesthesia [16]. Wei et al. reported that flumazenil antagonism of remimazolam might increase the incidence of PONV [7]. Although we did not specifically investigate PONV in our patients, the electronic patient records did not mention PONV in any of the five patients, and no antiemetic drugs were administered in the recovery room or on the ward. Interestingly, our extensive multimodal analgesic concept resulted in very low postoperative opioid use.

In rare cases (0.1-1%), the administration of flumazenil may lead to fear and palpitations. However, these side effects are transient and are most prominent when flumazenil is administered to awake patients rather than when used in asleep patients, as was the case in the current study design.

Due to the small sample size and retrospective study design, no strong conclusions can be drawn from our data. Furthermore, rather than focusing on individual variables, future studies should investigate whether our approach benefits overall recovery quality and the incidence of complications. More research involving larger groups of patients is warranted to verify whether the "switching and antagonizing" approach results in reduced and predictable extubation times and a more efficient workflow and reduces the risk of airway complications during and after extubation. This approach may be of particular interest to patients at increased risk of airway complications, such as those with difficult airways, bronchial hyperreactivity, or an elevated risk of aspiration.

## Conclusions

Our case series demonstrates that the "switch and antagonize" concept is feasible and resulted in a rapid and smooth emergence and recovery in five patients, free from excitation or hemodynamic and respiratory disturbances. This was achieved by switching from combining remimazolam, propofol, and sevoflurane to remimazolam as the sole hypnotic agent, combined with flumazenil. Additionally, postoperative opioid use was minimal, and there was no need for antiemetic medication. Whether the "switch and antagonize" protocol could shorten the time to extubation and improve recovery predictability and efficiency should be investigated further. Moreover, cost-effectiveness and further evaluation and fine-tuning of this concept are needed, with particular emphasis on its application in patients with risk factors such as difficult airways or an increased risk of aspiration. It appears promising for future clinical use, especially for vulnerable patient groups, but larger (case-control) studies are essential to fully understand this approach's potential.

## References

[1] Popat M, Mitchell V, Dravid R, Patel A, Swampillai C, Higgs A (2012). Difficult Airway Society Guidelines for the management of tracheal extubation. Anaesthesia.

[2] Benham-Hermetz J, Mitchell V (2021). Safe tracheal extubation after general anaesthesia. BJA Educ.

[3] He TY, Zhong RP, Zhong WB, Huang GM, Liu XC (2024). Effect of remimazolam on intra-operative hypotension: systematic review and meta-analysis of randomised controlled trials. Eur J Anaesthesiol.

[4] Yang C, Jiao J, Nie Y, Shao W, Zhang H, Huang S (2024). Comparison of the bispectral indices of patients receiving remimazolam and propofol for general anesthesia: a randomized crossover trial. Anaesth Crit Care Pain Med.

[5] Zhang S, Wang J, Ran R, Peng Y, Xiao Y (2022). Efficacy and safety of remimazolam tosylate in hysteroscopy: a randomized, single-blind, parallel controlled trial. J Clin Pharm Ther.

[6] Lee J, Kim DH, Ju JW, Nam K, Cho YJ, Jeon Y, Lee S (2024). Comparison of recovery profiles between total intravenous anaesthesia with propofol or remimazolam reversed with flumazenil in patients undergoing breast surgery: a randomised controlled trial. Eur J Anaesthesiol.

[7] Wei Y, Zhu M, Man Y, Xiao H, Dong G, Shi X, Ji F (2024). Clinical study of flumazenil antagonizing remimazolam on nausea and vomiting after gynecologic day surgery. Drug Des Devel Ther.

[8] Araki H, Inoue S (2023). Switching to remimazolam followed by flumazenil may be a promising combination for deep extubation. JA Clin Rep.

[9] Jeon S, Kim J, Karm MH, Kim JT (2024). Effect of converting from propofol to remimazolam with flumazenil reversal on recovery from anesthesia in outpatients with mental disabilities: a randomized controlled trial. BMC Anesthesiol.

[10] Wu Q, Xu F, Wang J, Jiang M (2023). Comparison of remimazolam-flumazenil versus propofol for recovery from general anesthesia: a systematic review and meta-analysis. J Clin Med.

[11] Koch R, Witkam R, van Eijk LT, Bruhn J (2024). Triple anesthesia: combining sevoflurane, propofol, and remimazolam for general anesthesia in a case series. Cureus.

[12] Yaraghi A, Golparvar M, Talakoub R, Sateie H, Mehrabi A (2013). Hypercapnic hyperventilation shortens emergence time from Propofol and Isoflurane anesthesia. J Res Pharm Pract.

[13] Sakata DJ, Gopalakrishnan NA, Orr JA, White JL, Westenskow DR (2007). Rapid recovery from sevoflurane and desflurane with hypercapnia and hyperventilation. Anesth Analg.

[14] Sakata DJ, Gopalakrishnan NA, Orr JA, White JL, Westenskow DR (2007). Hypercapnic hyperventilation shortens emergence time from isoflurane anesthesia. Anesth Analg.

[15] Toyota Y, Kondo T, Oshita K (2023). Remimazolam-based anesthesia with flumazenil allows faster emergence than propofol-based anesthesia in older patients undergoing spinal surgery: a randomized controlled trial. Medicine (Baltimore).

[16] Sato T, Mimuro S, Kurita T, Kobayashi M, Doi M, Katoh T, Nakajima Y (2022). Recall of extubation after remimazolam anesthesia with flumazenil antagonism during emergence: a retrospective clinical study. J Anesth.

